# A scenario for the emergence of protoviroids in the RNA world and for their further evolution into viroids and viroid-like RNAs by modular recombinations and mutations

**DOI:** 10.1093/ve/veab107

**Published:** 2022-01-15

**Authors:** Ricardo Flores, Beatriz Navarro, Pedro Serra, Francesco Di Serio

**Affiliations:** Instituto de Biología Molecular y Celular de Plantas, Consejo Superior de Investigaciones Científicas–Universidad Politécnica de Valencia, Ingeniero Fausto Elio s/n, Valencia 46022, Spain; Istituto per la Protezione Sostenibile delle Piante, Consiglio Nazionale delle Ricerche, Via Amendola 122/D, Bari 70126, Italy; Instituto de Biología Molecular y Celular de Plantas, Consejo Superior de Investigaciones Científicas–Universidad Politécnica de Valencia, Ingeniero Fausto Elio s/n, Valencia 46022, Spain; Istituto per la Protezione Sostenibile delle Piante, Consiglio Nazionale delle Ricerche, Via Amendola 122/D, Bari 70126, Italy

**Keywords:** origin of life, RNA evolution, rolling circle mechanism, hammerhead ribozyme, viroid-like RNA, satellite RNA

## Abstract

Viroids are tiny, circular, and noncoding RNAs that are able to replicate and systemically infect plants. The smallest known pathogens, viroids have been proposed to represent survivors from the RNA world that likely preceded the cellular world currently dominating life on the earth. Although the small, circular, and compact nature of viroid genomes, some of which are also endowed with catalytic activity mediated by hammerhead ribozymes, support this proposal, the lack of feasible evolutionary routes and the identification of hammerhead ribozymes in a large number of DNA genomes of organisms along the tree of life have led some to question such a proposal. Here, we reassess the origin and subsequent evolution of viroids by complementing phylogenetic reconstructions with molecular data, including the primary and higher-order structure of the genomic RNAs, their replication, and recombination mechanisms and selected biological information. Features of some viroid-like RNAs found in plants, animals, and possibly fungi are also considered. The resulting evolutionary scenario supports the emergence of protoviroids in the RNA world, mainly as replicative modules, followed by a further increase in genome complexity based on module/domain shuffling and combination and mutation. Such a modular evolutionary scenario would have facilitated the inclusion in the protoviroid genomes of complex RNA structures (or coding sequences, as in the case of hepatitis delta virus and delta-like agents), likely needed for their adaptation from the RNA world to a life based on cells, thus generating the ancestors of current infectious viroids and viroid-like RNAs. Other noninfectious viroid-like RNAs, such as retroviroid-like RNA elements and retrozymes, could also be derived from protoviroids if their reverse transcription and integration into viral or eukaryotic DNA, respectively, are considered as a possible key step in their evolution. Comparison of evidence supporting a general and modular evolutionary model for viroids and viroid-like RNAs with that favoring alternative scenarios provides reasonable reasons to keep alive the hypothesis that these small RNA pathogens may be relics of a precellular world.

## 1. Introduction

Viroids are a unique and enigmatic class of single-stranded ribonucleic acids (RNAs). Containing only ∼250–430 nucleotides (nts), they are able to replicate autonomously (i.e. in the absence of a coinfecting helper virus) in their hosts (restricted so far to higher plants) and to invade them systemically and, in some instances, cause disease. In addition to their small size, all viroids share: (1) a circular structure, (2) a high self-complementarity, leading to compact secondary structures, (3) replication through a rolling circle mechanism with only RNA intermediates, and, most importantly, (4) a lack of protein-coding ability (Diener 1971a,b, 2003; Gross et al. 1978; Flores et al. 2005, 2014, 2015; Hadidi et al. 2017; Steger and Riesner 2018; Navarro, Flores, and Di Serio 2021).

The lack of coding capacity, in particular, distinguishes viroids from RNA viruses, which in all cases (excepting some plant satellite viruses) encode at least the RNA-dependent RNA polymerase (RdRp), the only protein common to all RNA viruses and essential for their replication (Wolf et al. 2018; Krupovic, Dolja, and Koonin 2019). Moreover, while typical RNA viruses replicate in membranous cytoplasmic vesicles that are partly induced by viral-encoded proteins (den Boon, Diaz, and Ahlquist 2010), no such ‘viral factories’ have been observed in tissues infected by viroids. Viroids, in contrast, appear to be recognized, transcribed, and processed as endogenous cellular RNAs (Gross et al. 1978).

Several scenarios have been proposed to explain the origin of viroids (for a review, see Flores et al. 2014). Support for early suggestions that viroids might have come from ‘escaped’ introns (Diener 1981) or from transposable elements (Kiefer, Owens, and Diener 1983) has declined over time, mainly due to the lack of sequence identity between the genomes of viroids and their hosts (Branch and Dickson 1980; Zaitlin et al. 1980; Hadidi, Cress, and Diener 1981). While research on a plant satellite RNA has found some support for its host origin (Zahid et al. 2015), similar data have not been reported for viroid-like satellite RNAs of similar size. Consideration of a broader range of functional properties suggested that viroids (or, better, ‘protoviroids’ of reduced complexity) might instead have originated in the RNA world before the advent of DNA and proteins (Diener 1989; Flores et al. 2014). These include their small size, circularity, and compact secondary structure, which would afford resistance to nucleases and ultraviolet (UV) irradiation and, additionally, avoid the need for initiation and termination signals during replication; high G+C content, which would stiffen their secondary structure and diminish the effects of the low fidelity of primitive polymerases; the lack of protein-coding ability (the ribosome did not yet exist); and, most remarkably, the catalytic activity of some viroid RNAs. Free-living protoviroid RNAs would have adopted an intracellular mode of existence once cellular organisms appeared.

Such view of viroids as ‘survivors’ from the RNA world (Diener 1989; Flores et al. 2014) and the possible origin of all life on earth (Moelling and Broecker 2021) have been questioned, given the lack of a feasible evolutionary route explaining the presence of these postulated RNA ‘fossils’ only in angiosperms (∼200 million year old) but not in their prokaryote algal ancestors (∼3,500 million year old) (Chela-Flores 1994), leading the initial proponent to withdraw his backing (Diener 2016). In addition, theoretical simulations suggest that viroid-like replicons might have emerged *de novo* in plant or animal cells seeded by a pool of small RNAs providing a minimal combination of sequence and structural motifs eventually recognized by the cell replication machinery (Catalán et al. 2019). Alternatively, when seeded with DNA, *in vitro* assays with T7 phage RNA polymerase yield new replicating RNAs (Jain et al. 2020).

We believe that the ‘RNA world’ hypothesis, when compared with others, deserves further examination, in some ways, standing as the ‘default hypothesis’. To illustrate our reasoning, it is useful to consider a model for the evolution of RNA viruses recently proposed by Krupovic and Koonin (2017), which integrates features from the so-called ‘virus-first’ (viruses derived from precellular genetic elements) and the ‘escape’ (genes that got away from ribocells and eventually became encapsidated) models. Accordingly, modern viruses would be composed of two genetic modules: one involved in replication, originated in a ‘primordial world’ that existed prior to cells, and the other in virion assembly, with genes (like that encoding the typical capsid) captured from cells after they emerged in evolution. This modular model (Krupovic and Koonin 2017; Wolf et al. 2018; Krupovic, Dolja, and Koonin 2019) has been criticized because of (1) the lack of a reliable sequence alignment of the RdRp (Holmes and Duchêne 2019) and (2) the lack of consistency with models based on protein structure rather than on sequence (Nasir and Caetano-Anollés 2015; Mughal, Nasir, and Caetano-Anollés 2020). Nevertheless, the modular model provides deep insights into the appearance and diversification of major extant groups of RNA viruses. Irrespective of the evolutionary model adopted, viruses and their hosts are closely intertwined due to their using the same translation apparatus.

In contrast with RNA viruses, the emergence and evolution of viroids present fewer problems because viroids are considerably smaller in size and do not code for any protein. In fact, they are parasites of the host transcription machinery, and the virion assembly module is unnecessary. Furthermore, some of the viroids contain (encode) ribozymes, the key signature of a ‘primordial world’ and, more specifically, of an RNA world (Atkins, Gesteland, and Cech 2011). The model presented below also has a modular flavor, although of a different kind.

In developing our scheme for the origin and subsequent evolution of viroids, we have complemented phylogenetic reconstructions with both relevant molecular data (base composition and higher-order structure of the genomic RNA, type of rolling circle replication mechanism, and evidence of recombination of domains/modules) and available biological information (mostly host range but also transmission and interactions between coinfecting viroids or variants thereof). In doing so, we have followed an approach previously applied to RNA viruses (Wolf et al. 2018). Where appropriate, we have also incorporated in our scheme the features of some viroid-like RNAs found to infect plants and animals. The resulting picture portrays a plausible scenario for the emergence of protoviroids in the RNA world, with their further increase in complexity proceeding by the shuffling of modules/domains and by mutation. Modular evolution permits higher mutation rates and shorter evolutionary times, thus facilitating the emergence of complex structures otherwise impossible to select directly (Manrubia and Briones 2007). Although the scheme we present does not answer all major questions, some risks are necessary when rewinding the history of life back to more than 3500 million years.

## 2. Family *Avsunviroidae:* catalytic modules from the RNA world often fused to other modules of unknown provenance

**Figure 1.**
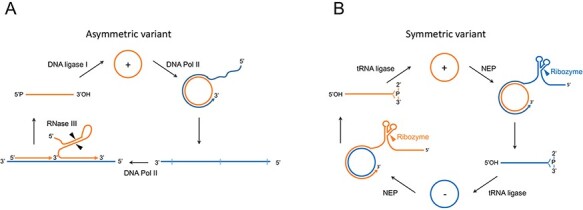
Rolling circle mechanism proposed for the replication of viroids. Members of the families *Avsunviroidae* (A) and *Pospiviroidae* (B) follow symmetric (two rolling circles) and asymmetric (one rolling circle) alternative versions of the mechanism, respectively. Orange and blue colors refer to (+) (by convention the most abundant *in vivo*) and (−) RNA polarities, respectively, with cleavage sites indicated by arrowheads. The enzymes and ribozymes catalyzing the replication steps are reported: the nuclear DNA Pol II and the plastid DNA-dependent NEP are redirected to transcribe RNA instead of DNA templates; the DNA ligase 1 is redirected to circularize RNA inestead of DNA substrates.

Members of this family adopt rod-like, quasi-rod-like, or multibranched secondary structures and replicate in plastids, mostly chloroplasts, through a symmetric double rolling circle mechanism in which the oligomeric RNA (+) and (−) intermediates self-cleave by hammerhead ribozymes (HHRs) (Hutchins et al. 1986; Prody et al. 1986) embedded in either polarity strand (Branch and Robertson 1984) (Fig. 1). Such viroids do not have a central conserved region (CCR) (McInnes and Symons 1991) as those of the family *Pospiviroidae* (type member, potato spindle tuber viroid, PSTVd) do. Of note, members of the *Avsunviroidae* infect a very narrow range of hosts (dicots), restricted to those in which each viroid was initially reported and to some closely related plant species. Below, relevant structural and biological features of these chloroplast replicating RNAs are considered in the frame of possible scenarios for their emergence and evolution.

### 2.1 Avocado sunblotch viroid: a rod-shaped RNA containing HHRs

**Figure 2.**
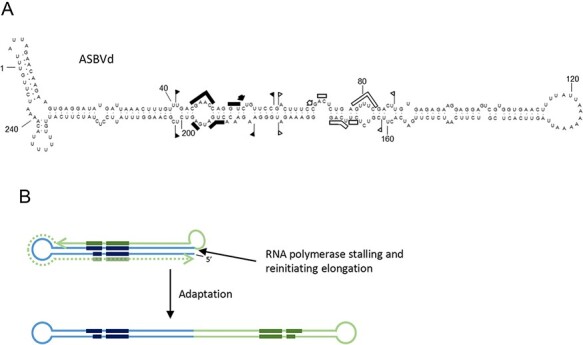
(A) Conformation adopted by ASBVd *in vivo* according to data from (López-Carrasco and Flores 2017a). The conserved nucleotides of HHRs and the respective cleavage sites are indicated by bars and arrows. Flags indicate the sequences forming the HHRs. Black and open symbols refer to (+) and (−) polarity, respectively. (B) Schematic representation of the origin of a protoviroid of ASBVd from a replicative module containing the HHR. The RNA polymerase stalls at the 3ʹ end of the nascent transcript (light blue line) containing the conserved domains of an HHR (dark blue segments) and reinitiates elongation copying back the nascent transcript up to its 5ʹ terminal (green line), thus generating a structure similar to the original one that may originate the right half of a rod-like structure by mutation and adaptation. Interestingly, the final disposition of the conserved hammerhead domains in such a rod-like structure is the same found in the current ASBVd genome. Additional jumping-reinitiation steps could have add some short sequence forming the additional short HPs observed in the secondary structure of ASBVd.

Avocado sunblotch viroid (ASBVd), type member of the family *Avsunviroidae* and of the genus *Avsunviroid* (vernacular name, avsunviroid) (Di Serio et al. 2018a), is one of the smallest viroids (247 nt) and the only one with a base composition rich in A + U (62 per cent) (Symons 1981). ASBVd RNA folds into a rod-like conformation *in silico* (Symons 1981), *in vitro* (Giguère et al. 2014a), and *in vivo* (López-Carrasco and Flores 2017a) (Fig. 2A). Its replication in the chloroplast proceeds through a symmetric double rolling circle (Daròs et al. 1994; Navarro, Daròs, and Flores 1999) and is mediated by a nuclear-encoded DNA-dependent RNA polymerase (NEP) redirected to transcribe RNA templates (Navarro and Flores 2000; Navarro, Vera, and Flores 2000), *cis-*acting HHRs (Hutchins et al. 1986), and a nuclear-encoded tRNA ligase (Nohales et al. 2012b) (Fig. 1). The ASBVd HHRs are thermodynamically unstable, particularly that of the (+) strand and operate as double HHRs only during the transcription of the oligomeric RNAs (Forster et al. 1988; Davies, Sheldon, and Symons 1991), thus ensuring self-cleavage of the latter and not of the final monomeric circular RNAs.

**Figure 3.**
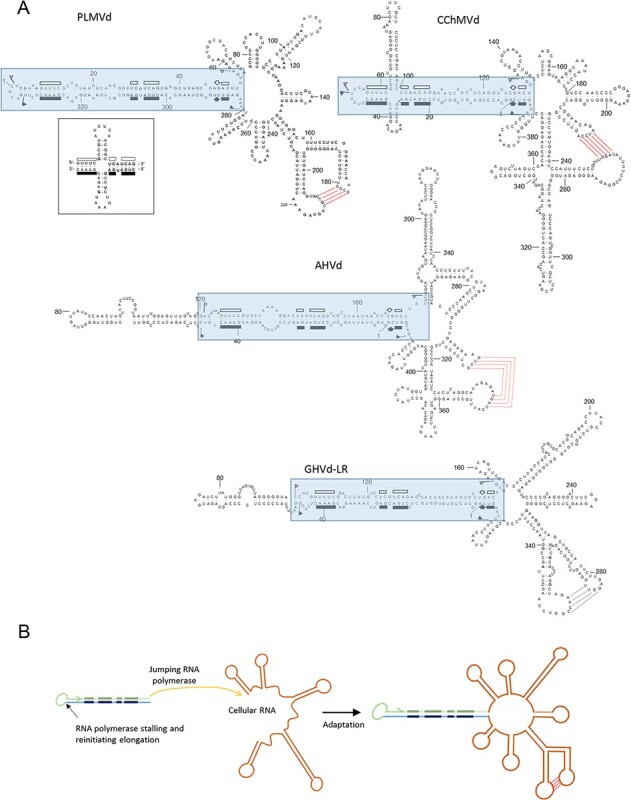
(A) Branched conformation proposed for PLMVd, CChMVd, AHVd, and GHVd-LR. Boxed sequences correspond to the possible replicative module derived from an ancestor protoviroid. Symbols are as reported in the legend to Fig. 2. Inset: alternative cruciform structure of the hammerhead arm proposed for some PLMVd sequence variants. (B) Schematic representation of the origin of an ancestor protoviroid of pelamoviroids from a replicative module containing the HHR: the RNA polymerase stalls at the 3ʹ end of the nascent transcript (light blue line) containing the conserved domains of an HHR (dark blue segments) and reinitiates elongation copying back the nascent transcript up to its 5ʹ terminal (green line). In this protoviroid, likely generated in the RNA word, the final disposition of the conserved hammerhead domains is the same of the current hammerhead arm found in the genome of all pelamoviroids. It is proposed that within the host cell, the polymerase replicating such a protoviroid after stalling at the 3ʹ terminal of the nascent RNA jumped to a host mRNA and reinitiated transcription copying such an RNA. Mutation and adaptation steps would have finally generated the current pelamoviroids adapted to their hosts. This scheme is consistent with the presence of a similar hammerhead arm in all pelamoviroids and with the presence of largely divergent sequences in most of the rest of their genomic RNAs.

How might ASBVd have emerged? It is unlikely that even a small viroid such as ASBVd would have appeared at a stroke. Close inspection of the rod-shaped structure (Fig. 2A) shows that half of this structure may well derive from the other. If (+) strands synthesis initiates around Position 180 and then proceeds toward Position 60 (encompassing the left half of the ‘rod’), with the RNA polymerase then stalling and reinitiating elongation at the 3ʹ-end of the nascent transcript and copying it back up to Position 180, a structure similar to the right half of the ‘rod’ is produced from which, by mutation and adaptation, ASBVd could have evolved overtime (Fig. 2B). Early studies have suggested the involvement of ‘jumping’ RNA polymerases in the genesis of viroids and viroid-like RNAs (Keese and Symons 1985; Forster and Symons 1987), and the whole ASBVd RNA may have originated by intramolecular recombination resulting from discontinuous transcription. Such a scheme would explain why the sequences forming each HHR are found in the upper and lower strands of the rod-shaped structure of ASBVd but not only in the upper or lower strands of a rod-shaped module portion of the branched conformations adopted by the other members of the family *Avsunviroidae* (see below, Fig. 3).

### 2.2 Pelamoviroids may have been originated from protoviroids that captured a multibranched domain

Peach latent mosaic viroid (PLMVd, 338 nt) is the type member of the genus *Pelamoviroid* (vernacular name, pelamoviroids). Its discovery (Hernández and Flores 1992) established on firm ground the creation of a second family (*Avsunviroidae*) for grouping those viroids not included in the first one (*Pospiviroidae*). *In silico* (Hernández and Flores 1992; Ambrós et al. 1998), *in vitro* (Bussière et al. 2000; Dubé et al. 2010, 2011; Moreno et al. 2019) and *in vivo* (Ambrós et al. 1998; Ambrós, Hernández, and Flores 1999), PLMVd adopts a complex secondary structure containing a rod-like domain fused to a multibranched domain stabilized by a kissing-loop interaction in the (+) but not in the (−) strand (Bussière et al. 2000; Dubé et al. 2010) (Fig. 3). The HHRs of PLMVd are thermodynamically stable and their sequences face each other in the rod-like catalytic domain and are mostly base-paired (Hernández and Flores 1992). Because this catalytic domain contains the self-cleavage/ligation sites, as well as the initiation sites of both (+) and (−) strands (Delgado et al. 2005), it can be regarded as a sort of ‘replicative module’ or ‘protoviroid’.

A plausible scenario for PLMVd origin begins with the catalytic domain (a long hairpin (HP) of ∼100 nt) emerging in the RNA world as the result of template switching by a ‘jumping’ RNA polymerase. Having transcribed the lower strand of this domain, the polymerase would have turned back and resumed transcription using the recently synthesized RNA as a template, thereby producing both strands connected by a small loop. This mechanism would explain why the catalytic domain is essentially self-complementary. Moreover, because each strand is itself self-complementary, the catalytic domain can also adopt an alternative cruciform structure (Fig. 3A, inset) (Ambrós et al. 1998; Ambrós, Hernández, and Flores 1999; Dubé et al. 2011). Such a cruciform structure could also be the result of HP capture, which may have played a role in the formation of larger RNAs (see below). Even under current conditions, PLMVd is able to capture HPs, like that of 12–13 nt involved in peach calico disease (an extreme albinism) (Malfitano et al. 2003; Rodio et al. 2006, 2007; Navarro et al. 2012).

Altogether the size of the RNA to be synthesized nonenzymatically in the primordial scenario would be reduced to less than half the size of the ∼100-nt catalytic domain. This domain (or protoviroid) would then have eventually fused to the multibranched domain, assuming some evolutive advantage for the resulting recombinant; for example, intracellular or intercellular movement. Thus, the multibranched ∼230-nt domain, stabilized by a kissing-loop interaction, might have emerged at a later stage in the cellular world. There is experimental evidence, apart from that obtained for ASBVd, that NEP also catalyzes the polymerization of PLMVd strands in plastids (Rodio et al. 2007). The high mutation frequency observed in natural populations of PLMVd and, particularly, in the progeny resulting from artificial inoculations with cloned single variants (Ambrós et al. 1998; Ambrós, Hernández, and Flores 1999; Pelchat et al. 2000; Glouzon et al. 2014) indicates that PLMVd propagates in its host (peach) as complex populations of closely related variants (quasispecies) (see for review Flores et al. 2014). This finding, together with maintenance of the infectivity in artificial mutants with one or a few nt changes (Malfitano et al. 2003; Rodio et al. 2006, 2007; Navarro et al. 2012; Delgado et al. 2019), suggested a high mutation rate. Such issue was tackled with a more amenable experimental system formed by another pelamoviroid, chrysanthemum chlorotic mottle viroid (CChMVd, Navarro and Flores 1997), infecting naturally a herbaceous host.

CChMVd (399 nt) shows clear similarities with PLMVd, including a catalytic domain of about 130 nt, with stable HHRs in either polarity strand. The secondary structure predicted initially for CChMVd *in silico* (Fig. 3) should be essentially similar to that existing *in vivo*. In fact, analyses of natural covariations in CChMVd nt sequence showed that their occurrence preserves specific stems, supporting the role of selection in wiping out *in vivo* nonviable variants in which these specific stems are disrupted by single substitutions (Navarro and Flores 1997). The catalytic domain adopts a cruciform structure, with one of the branches enlarged (Fig. 3), possibly due to an HP capture. Moreover, a predicted kissing-loop interaction in the multibranched domain of the CChMVd (+) strand (Fig. 3), similar to that of PLMVd (Bussière et al. 2000), was later found to be critical for the *in vitro* folding and *in vivo* viability of CChMVd (Gago, de la Peña, and Flores 2005). The mutation rate of this viroid (1/400 nt transcribed), one of the highest documented for any biological entity (Gago et al. 2009) and reminiscent of those predicted for primitive replicons in the RNA world, most likely results from transcription by a proofreading-deficient single-subunit NEP redirected to accept RNA as template rather than its native DNA (Navarro, Vera, and Flores 2000; Rodio et al. 2007).

Apple hammerhead viroid (AHVd, 434 nt), initially identified as a viroid-like RNA by RNA-seq and a computational algorithm for discovering circular RNAs (Zhang et al. 2014), was later shown to be infectious and, thus, a *bona fide* viroid (Serra et al. 2018). AHVd shares clear similarities with PLMVd and CChMVd, including a rod-like catalytic domain of about 180 nt, with stable HHRs in either polarity strand, and a predicted kissing-loop interaction in the (+) strand stabilizing the multibranched domain (Fig. 3). Capture of extra sequences by AHVd and CChMVd may explain their larger sizes with respect to PLMVd.

Grapevine hammerhead viroid-like RNA (GHVd-LR, 375 nt; Wu et al. 2012) does not yet fulfill the criteria to be considered a genuine viroid because bioassays to verify its autonomous infectivity and (possible) pathogenicity are lacking (Di Serio et al. 2018b). However, its predicted secondary structure, a rod-like catalytic domain of about 160 nt, with stable HHRs in either polarity strand and a predicted kissing-loop interaction stabilizing the multibranched domain of the (+) strand, suggests that GHVd could be another pelamoviroid (Fig. 3).

While the three known pelamoviroids exhibit low sequence identity (ranging from 15 to 38 per cent; Chiumenti et al. 2021), important structural features—a catalytic domain fused to a multibranched domain stabilized by a kissing-loop interaction in the (+) strands—are highly conserved. This molecular architecture strongly suggests convergent evolution toward a similar higher-order structure, which is the ‘phenotype’ of viroids. While the catalytic domains have the features consistent with a common origin in the RNA world, the multibranched domains do not, and they may have emerged later on. The absence of a conserved primary structure makes it difficult to identify a common ancestor for the pelamoviroid multibranched domains. However, the higher-order structure of this domain is maintained among the members of the genus and residues involved in the kissing-loop interaction are separated by a stretch of 30–37 nt in all instances.

### 2.3 Eggplant latent viroid has a central replicative module embedded in a quasi-rod-like conformation

**Figure 4.**
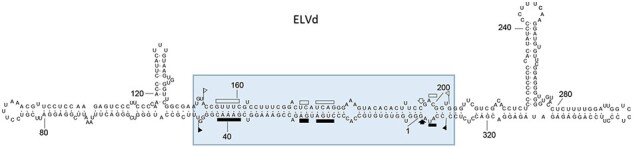
Semibranched conformation proposed for ELVd. The central catalytic region of ELVd, which contains the conserved domains of the HHRs of both polarity strands, can be considered as a replicative module derived from a protoviroid that later captured the RNA sequences now forming the right and left terminal domains. Symbols are as reported in the legend to Fig. 2.

Eggplant latent viroid (ELVd, 334 nt), the type member of the genus *Elaviroid* (vernacular name, elaviroid), adopts a quasi-rod-like conformation *in silico* (Fadda et al. 2003a), *in vitro* (Giguère et al. 2014a; Moreno et al. 2019), and *in vivo* (López-Carrasco et al. 2016), with bifurcations at both terminal domains (Fig. 4). Studies with ELVd, which can form in its (+) and (−) strands stable HHRs with long helices I, support that natural ribozymes of this class have been evolutionarily selected to function cotranscriptionally in the course of replication (Carbonell et al. 2006). In contrast, the sequences forming the HHRs are catalytically inactive in monomeric ELVd RNAs because they are opposed and mainly base-paired each other within the central domain of the quasi-rod-like conformation (Fadda et al. 2003a) (Fig. 4). Such a conformation most likely facilitates the circularization of the monomeric linear strands catalyzed by a chloroplastic tRNA ligase (Nohales et al. 2012b). A possible evolutionary scenario would have the central (rod-like) catalytic domain emerging in the RNA world and, only later, capturing the HPs that form the two terminal domains. Mutation of captured sequences would also have played a role. In this respect, high-fidelity ultra-deep sequencing to compare side-by-side data from a common host (eggplant) revealed that the mutation rate of ELVd is several fold higher than that of PSTVd (family *Pospiviroidae)* (López-Carrasco et al. 2017). However, PSTVd mutation rate was recently calculated to be higher in its natural host tomato (Wu and Bisaro 2020).

## 3. Family *Pospiviroidae:* uncertain origin of the ancestral protoviroid but clear subsequent diversification

Members of this family adopt a rod-like or quasi-rod-like secondary structure and replicate in the nucleus through an asymmetric single rolling circle mechanism in which only the oligomeric (+) RNA intermediates are processed into their monomeric circular form (Fig. 1) (Ishikawa et al. 1984; Branch, Benenfeld, and Robertson 1988; Daròs and Flores 2004). Rather than the HHRs typical of the *Avsunviroidae*, these viroids have a CCR that plays a major role in replication (Gas et al. 2007). As discussed in the next sections, several structural features of members of all the genera in family *Pospiviroidae* are consistent with a modular evolution.

### 3.1 Role of recombination and point mutations in the evolution of PSTVd and closely related pospiviroids

**Figure 5.**
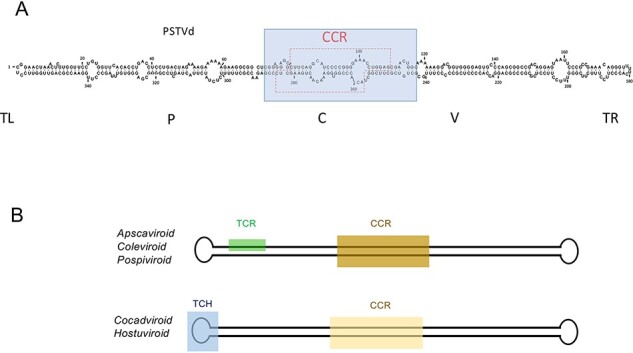
(A) The central domain (containing the CCR, boxed by the broken red line) of PSTVd and by extension of all members of the family *Pospiviroidae*, can be considered as a replicative module (blue box), likely derived from a protoviroid generated in the RNA world, that may have originated the current viroids through discontinuous transcription mediated by a jumping RNA polymerase and by further adaptation to the respective hosts. The five domains reported in PSTVd (Keese and Symons 1985) are indicated: TL: terminal left; P, pathogenic; C, central; V, variable; TR, terminal right. (B) Conserved domains (TCR, TCH, and CCR) shared by members of the family *Pospiviroidae* are reported. Members of the genera *Apscaviroid, Coleviroid* and *Pospiviroid* generally contain the TCR, while those of the genera *Cocadviroid* and *Hostuviroid* generally contain the TCH.

PSTVd (359 nt), the type member of the family *Pospiviroidae* and of the genus *Pospiviroid* (vernacular name, pospiviroid), was the first viroid discovered (Diener 1971a, 1972) and completely sequenced (Gross et al. 1978). PSTVd RNA folds into a rod-like conformation *in silico* (Gross et al. 1978), *in vitro* (Sogo, Koller, and Diener 1973; Riesner et al. 1979; Giguère, Adkar-Purushothama, and Perreault 2014b; Moreno et al. 2019), and *in vivo* (López-Carrasco and Flores 2017b). Within this conformation, five domains have been identified: central (containing the CCR), pathogenicity (with an A-rich motif in the upper strand present also in members of the other genera in the family *Pospiviroidae)*, variable, terminal left, and terminal right (Keese and Symons 1985) (Fig. 5A). As in the case of ASBVd, PSTVd accumulates *in planta* as a free RNA (Diener 1971b; López-Carrasco and Flores 2017b). PSTVd, and to a lesser extent citrus exocortis viroid (CEVd, 371 nt) (Semancik and Weathers 1972), served for most of the initial research on viroids. As shown in Fig. 1, PSTVd replication takes place in the nucleus through an RNA-RNA asymmetric single rolling circle mechanism (Grill and Semancik 1978; Spiesmacher et al. 1983; Branch, Benenfeld, and Robertson 1988; Feldstein, Hu, and Owens 1998; Daròs and Flores 2004). The infecting monomeric circular (+) RNA is repeatedly transcribed by the DNA-dependent RNA polymerase II (Pol II), redirected to accept RNA templates (Flores and Semancik 1982; Schindler and Mühlbach 1992) and assisted by transcription factor IIIA-7ZF (Wang et al. 2016; Jiang et al. 2018; Dissanayaka Mudiyanselage and Wang 2020). The resulting oligomeric (−) RNAs serve as template for the synthesis of complementary (+) strand oligomers, which are cleaved by an RNase of class III (Gas et al. 2007) and, then, circularized by the DNA ligase 1 redirected to accept RNA substrates (Nohales, Flores, and Daròs 2012a). The cleavage step relies on a metastable conformation adopted by oligomeric (+) strands during replication in which the upper CCR strand and the flanking imperfect repeats forming the so-called HP I (Riesner et al. 1979) play a major role. In contrast, ligation depends on both CCR strands (Gas et al. 2007).

From an evolutionary perspective, the central domain (∼90 nt) can be regarded as a protoviroid because it contains structural elements that direct two of the three replication steps, as well as the corresponding cleavage/ligation site. There is also evidence for mechanisms resulting in the enlargement of pospiviroid genomes, such as a simple duplication in the left terminal domain of PSTVd resulting from discontinuous transcription by a jumping RNA polymerase (Keese and Symons 1985), as well as certain CEVd variants isolated from tomato (Semancik et al. 1994) and eggplant (Fadda et al. 2003b). Despite containing either a 92-nt or 96-nt duplication within the variable and right terminal domains, these enlarged RNAs retain a predicted rod-like conformation. Variants with and without the 96-nt duplication coexist in eggplant (Fadda et al. 2003b), suggesting that the enlargement may be driven by selective pressure(s).

At least one example of genome reduction has also been reported, that is, an *in vitro*-generated noninfectious PSTVd mutant containing a 9-nt deletion has been observed to evolve into an infectious RNA *in vivo* (in tobacco) via the appearance of a complementary 9-nt deletion that restores the rod-shape secondary structure, thus highlighting its functional relevance (Wassenegger, Heimes, and Sänger 1994). Finally, there are naturally occurring chimeric viroids, like columnea latent viroid (CLVd, 370 nt), in which the CCR characteristic of pospiviroids has been replaced by that of hop stunt viroid (HSVd, genus *Hostuviroid,* see below), most likely as a result of intracellular RNA recombination between HSVd and one or more pospiviroids known to coinfect common host plants (Hammond, Smith, and Diener 1989).

In addition to the major modular events resulting in new chimeric viroids, the low fidelity of the Pol II during replication of PSTVd and other pospiviroids contributes to the propagation of these viroids as mixtures of closely related variants or quasispecies (Codoñer et al. 2006; Flores et al. 2014). Minor changes accumulate during adaptation to new hosts (Semancik et al. 1993; Bernad, Duran-Vila, and Elena 2009), thereby explaining the emergence of complex populations of PSTVd variants in tomato inoculated with PSTVd-cDNAs (Góra-Sochacka et al. 1997). In a natural context, PSTVd, CEVd, and other pospiviroids are widespread in asymptomatic solanaceous ornamentals, from where they have jumped to horticultural crops (Navarro et al. 2009; Verhoeven et al. 2010). Eventually, some viroid variants adapted to new hosts may evolve into new species.

### 3.2 Cocadviroids provide further evidence of discontinuous transcription by jumping RNA polymerase

Coconut cadang-cadang viroid (CCCVd, 247 nt), the type member of the genus *Cocadviroid,* was identified in coconut palm (Randles 1975; Randles and Hatta 1979) and predicted to adopt a rod-like conformation *in silico* (Haseloff, Mohamed, and Symons 1982) with five domains and a CCR related to that of PSTVd (Keese and Symons 1985). Members of this genus lack the terminal conserved region (TCR) that is only present in viroids larger than ∼300 nt but do have a terminal conserved hairpin (TCH) (Puchta, Ramm, and Sänger 1988; Flores, Di Serio, and Hernández 1997). As shown in Fig. 5B, these two motifs appear mutually exclusive (Flores, Di Serio, and Hernández 1997). Infected tissue also contains other 287-, 296-, 297-, and 301-nt enlarged forms of CCCVd, whose predicted rod-like conformations contain 41-, 50-, and 55-nt sequence duplications derived from the terminal right domain of the 247-nt viroid RNA (Haseloff, Mohamed, and Symons 1982). These enlarged forms may result from discontinuous transcription by a jumping RNA polymerase (Keese and Symons 1985). CCCVd is essentially restricted to coconut and oil palms (monocots), while coconut tinangaja viroid (254 nt) (Keese, Osorio-Keese, and Symons 1988), a close relative of CCCVd, has been only detected in coconut palms. The two other cocadviroids, hop latent viroid (256 nt) (Pallás, Navarro, and Flores 1987; Puchta, Ramm, and Sänger 1988) and citrus bark cracking viroid (CBCVd, 284 nt) infect a common host (hop), although CBCVd was initially reported in citrus (Duran-Vila et al. 1988; Puchta et al. 1991).

Two points of note in an evolutionary context. First, transcripts from the cloned central domain of CCCVd have been reported to self-cleave after denaturation with methylmercuric hydroxide followed by incubation with spermidine (Liu and Symons 1998). The site of self-cleavage maps to the lower CCR strand and generates 5ʹ-hydroxyl and possibly 2ʹ,3ʹ-cyclic phosphodiester termini, as those resulting from the *in vitro* self-cleavage of members of the family *Avsunviroidae* and some viroid-like satellite RNAs (see below). Sequences around the CCCVd self-cleavage site cannot form the HHR or HP ribozyme structures characteristic of other small self-cleaving plant RNAs, however (Symons 1997). This finding is intriguing because, if confirmed, it would establish a link between members of the two viroid families and with the RNA world. And second, CBCVd is a chimeric viroid that most likely originated by recombination of two other viroids, known to coinfect citrus. Indeed, CBCVd contains the terminal left domain of HSVd and the central and terminal right domains of CEVd. From citrus, CBCVd could have jumped into hop where it causes a severe disease (Jakse et al. 2015).

### 3.3 The type member of hostuviroids contains a fossil footprint of hammerhead structure and shows an unusual wide host range

HSVd (297 nt), the type member of the genus *Hostuviroid,* was initially identified and sequenced in hop (Sasaki and Shikata 1977; Ohno et al. 1983). Its natural host range was later found to be the widest of any viroid and in only some of its herbaceous and woody crops does it incite disease. HSVd was used as a model to show the involvement of Pol II in the replication of nuclear viroids (Mühlbach and Sänger 1979) and to study the systemic movement and pathogenesis of viroids (reviewed by Marquez‐Molins, Gomez, and Pallas 2021). *In silico,* HSVd folds into a rod-like conformation with five domains and a CCR related to PSTVd but distinct from the CCCVd subclass (Ohno et al. 1983; Keese and Symons 1985). Instead of a TCR, HSVd contains a TCH (Flores, Di Serio, and Hernández 1997) (Fig. 5B).

From an evolutionary perspective, two aspects of HSVd structure deserve a comment. First, sequences within the terminal right domain of HSVd can fold into a hammerhead-like structure. Because these sequences are strictly conserved in all the sequence variants examined thus far, Amari et al. (2001) suggested that the hammerhead-like structure could represent an evolutionary link between HSVd and members of the family *Avsunviroidae*; in other words, a sort of ‘fossil’ footprint left behind during the transition of a self-cleaving into a non-self-cleaving viroid RNA. And second, the unusual wide host range of HSVd, the reason of which is presently unknown, may have favored the emergence of chimeric viroids by intracellular RNA recombination catalyzed by a jumping RNA polymerase in plants coinfected by another viroid(s). As in the case of CLVd described above, dahlia latent viroid (342 nt) (Verhoeven et al. 2013) contains the CCR of HSVd and the TCR of pospiviroids, two of which (PSTVd and chrysanthemum stunt viroid) naturally infect dahlia.

### 3.4 Apscaviroids occur as complex mixtures of coinfecting viroids likely favoring the emergence of chimeric viroids

Apple scar skin viroid (330 nt), the type member of the genus *Apscaviroid*, folds into a rod-shaped conformation *in silico* containing five domains, a CCR distinct from that of PSTVd (Keese and Symons 1985; Hashimoto and Koganezawa 1987) and the TCR (Flores, Di Serio, and Hernández 1997) (Fig. 5B). As many as ten apscaviroids together with seven additional candidate species have been reported (Chiumenti et al. 2021). Apscaviroids are particularly prevalent in apple, grapevine, and citrus, in the latter case forming complex mixtures with different viroids (including a pospiviroid, CEVd, and a hostuviroid, HSVd) coinfecting the same host (Duran-Vila et al. 1988). These natural coinfections most likely provide the conditions for the emergence of chimeric viroids like CBCVd (see above) and grapevine yellow speckle viroid 2 (363 nt), which contains a 69-nt sequence in the terminal left domain almost identical to the corresponding domain of a pospiviroid (tomato planta macho viroid) (Koltunow and Rezaian 1989). As observed for CEVd, the host influence on the genetic stability of viroid populations has been documented for the apscaviroid citrus dwarfing viroid (Tessitori et al. 2013).

### 3.5 Several coleviroids are chimeric RNAs deriving from recombination events

Coleus blumei viroid 1 (CbVd-1, 248 nt), the type member of the genus *Coleviroid*, was initially identified (Fonseca et al. 1989) and sequenced in ornamental coleus species (Spieker et al. 1990). CbVd-1 also naturally infects four species of the family Labiatae (Ishiguro, Sano, and Harada 1996). CbVd-1 folds into a rod-like conformation *in silico* with five domains and a CCR distinct in sequence from those of members of the other viroid genera (Spieker et al. 1990; Flores, Di Serio, and Hernández 1997). Its terminal left HP resembles in structure but only partly in sequence, the TCH present in all members of the *Pospiviroidae* containing less than 300 nt.

Some coleus plants are naturally coinfected by CbVd-1 and by two other members of its genus, coleus blumei viroid 2 and 3 (CbVd-2 and CbVd-3, respectively). CbVd-2 is composed of two blocks of sequences, one identical to the right-hand portion of the rod-shaped structure of CbVd-1 and the other identical to the left-hand portion of the rod-shaped structure of CbV-3, with sharp demarcation boundaries between the two blocks of sequences. Therefore, CbVd-2 most likely emerged by recombination between CbVd-1 and CbVd-3 coinfecting the same plant (Spieker 1996). Other chimeric viroids of this genus, such as coleus blumei viroid-5, -6, and -7, very likely derived from recombination events involving other combinations of coinfecting coleus viroids (Hou et al. 2009a,b; Smith et al. 2021).

## 4. Phylogenetic analysis is consistent with a monophyletic origin for members of the family *Pospiviroidae*

**Figure 6.**
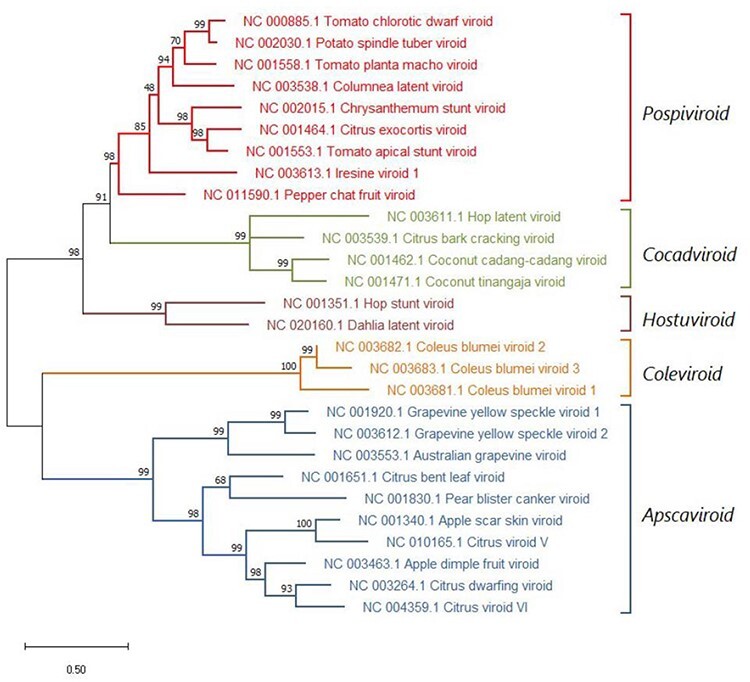
Maximum likelihood phylogenetic tree inferred with the reference variants of the species currently classified by International Committee on Taxonomy of Viruses (ICTV) in the five genera of the family *Pospiviroidae* and of the viroids yet unclassified (in red). Bootstrap values (generated by 1,000 replicates) are shown next to the branches. The tree is drawn to scale, with branch lengths measured in the number of substitutions per site.

The presence of conserved CCR and TCR or TCH regions/motifs located in similar positions within the rod-shaped secondary structure makes it feasible to align the corresponding sequences and to infer a phylogenetic reconstruction, supported in most branches by high bootstrap values, in which members of each genus group together (Fig. 6) (Di Serio et al. 2021). In this context, the major forces driving viroid speciation are point mutations resulting from adaptation to new hosts and, especially, recombination (via a jumping RNA polymerase) between viroids coinfecting the same host. In contrast, earlier phylogenetic reconstruction including members of both viroid families (and even viroid-like satellite RNAs) (Elena et al. 1991, 2001) should be regarded with care because of the lack of extensive sequence similarity (Jenkins et al. 2000, and see above).

## 5. Viroid-like catalytic RNAs from plants and animals: support for a link with the family *Avsunviroidae*

Resembling members of the family *Avsunviroidae,* some viroid-like RNAs display catalytic activity mediated in most instances by HHRs but also by another two classes of ribozymes. These viroid-like RNAs, some of which have a DNA counterpart, are biologically different from viroids and each other. Present not only in plants but also in animals and possibly in fungi, they offer the opportunity to compare their possible origin and evolution with that of viroids.

### 5.1 Plant viroid-like satellite RNAs may contain different ribozymes

The term ‘viroid-like satellite RNA’ refers to certain viral satellite RNAs, which are similar to viroids in size (∼220–370 nt), circularity, compact secondary structure, rolling circle replication, and generally lack of protein-coding ability (Schneider 1969; Roossinck, Sleat, and Palukaitis 1992; Symons and Randles 1999). Unlike viroids, however, viroid-like satellite RNA replication is not autonomous but depends upon an RdRp encoded, at least in part, by a specific coinfecting helper RNA virus. The circular or linear forms of the viroid-like satellite RNAs are specifically encapsidated by the coat protein of the helper virus, thereby allowing systemic movement and horizontal transmission. Moreover, ribozymes contained by viroid-like satellite RNAs may be of either the HHR (as in the satellite RNAs of sobemoviruses) or HP (as in the satellite RNAs of nepoviruses) type (Buzayan, Gerlach, and Bruening 1986). Although PSTVd (family *Pospiviroidae)* has also been found occasionally trans-encapsidated by a luteovirus coat protein, with the resulting particles being transmitted plant-to-plant by an aphid (Querci et al. 1997; Syller, Marczewski, and Pawłowicz 1997), such process is probably nonspecific and, hence, different from that observed in plant viroid-like satellite RNAs in which replication and encapsidation by the helper virus might be even linked.

Given the strong similarities between viroid-like satellite RNAs and viroids (particularly of the family *Avsunviroidae),* a common origin in the RNA world has been proposed (Diener 1989; Elena et al. 1991; Flores et al. 2014). Particularly striking is the fact that viroid-like satellite RNAs of nepoviruses contain an HHR in the (+) strand (by convention the most abundant, encapsidated strand), which catalyzes self-cleavage of the oligomeric intermediates (Prody et al. 1986), and an HP ribozyme in the (−) strand, which catalyzes not only self-cleavage of the oligomeric intermediates but also circularization of the resulting monomeric linear strands (Buzayan, Gerlach, and Bruening 1986). These ribozymes map to different RNA modules (Passmore et al. 1995), suggesting their independent capture in the same molecule, possibly in the RNA world. Interestingly, it has been recently proposed that the HHR and the bifunctional HP ribozyme may share evolutionary history in an RNA word, with the latter evolving from the former through mutational walks (Das Gupta, Nykiel, and Piccirilli 2021). Intriguingly, bioinformatic searches have not identified HP ribozymes apart from those found in viroid-like satellite RNAs of nepoviruses, despite such ribozymes being of similar complexity as HHRs and clearly more complex than those of typical deltaviruses, both of which are widely distributed along the tree of life (see below).

### 5.2 Viroid and viroid-like satellite RNAs infecting fungi?

A recent report that typical members of both viroid families are able to infect and induce symptoms in filamentous phytopathogenic fungi (Wei et al. 2019) was quite unexpected. Moreover, the seven viroids tested were also found to infect *Saccharomyces cerevisiae*, extending a proposal made previously for ASBVd (Delan-Forino, Maurel, and Torchet 2011). However, such proposal has been questioned (Serra et al. 2020) on several basis: (1) absence of proper controls, (2) lack of reproducibility of some results, and (3) difficulty in reconciling these observations with those from ASBVd-infected avocado, in which RNA polymerization, self-cleavage, and circularization of both viroid strands occur in chloroplasts, an organelle not present in filamentous fungi and yeasts. Furthermore, the sequence variability detected in the (presumed) viroid progeny from fungi consisted essentially of disperse point mutations (Wei et al. 2020) and not of the major and specific sequence changes expected to accumulate in the adaptation of viroid populations to a new fungal host. Thus, a critical reassessment, using a second detection approach and additional controls, is needed to confirm the ability of viroids to infect and cause disease in fungi (Serra et al. 2020).

The only known example of viroid-like RNAs that could conceivably infect fungi naturally are two small circular RNAs, cscRNA1 (∼400 nt) and its derivative cscRNA2 (∼375 nt), isolated from trees expressing cherry chlorotic rusty spot (CCRS) disease. The sequence variability detected does not affect the HHRs embedded in either polarity strand, arguing that they mediate cscRNA replication. However, cscRNA1 seems more closely related to viroid-like satellite RNAs than to viroids, suggesting that one of the dsRNA mycoviruses also associated with CCRS disease may serve as a helper virus (Di Serio et al. 1997, 2006). Further supporting such a scenario, viroid-like RNAs similar to cscRNA1, and always in close association with similar mycoviral dsRNAs, have been identified in different cherry cultivars growing in Spain and Italy (Minoia et al. 2014).

Recently, a novel viroid-like RNA, tentatively named fig hammerhead viroid-like RNA (FHVd-LR) and containing HHRs in both polarity strands, has been detected in fig trees growing in Hawaii (Olmedo-Velarde et al. 2020). The exact biological nature of FHVd-LR has not yet been determined, but as for cscRNAs, attempts of infecting fig seedlings by grafting or slash inoculation of purified RNA preparations or *in vitro* head-to-tail dimeric transcripts of the viroid-like RNA have so far been unsuccessful. Therefore, rather than being directly associated with the plant, the possibility that this viroid-like RNA may depend upon a virus infecting a fungus or another organism transiently associated with fig has been proposed as a possible alternative (Olmedo-Velarde et al. 2020).

**Figure 7.**
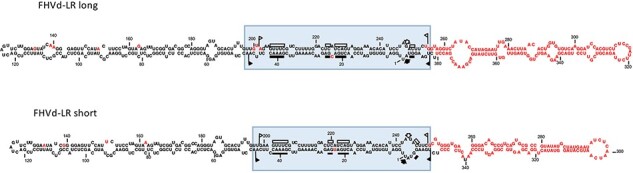
Rod-like conformation proposed for the short and the long hammerhead viroid-like RNAs associated with fig trees (FHVd-LR short and FHVd-LR long, respectively). Nucleotides differing in the two RNAs are indicated in red. The central catalytic region of FHVd-LRs (boxed) contains the conserved domains of the HHRs of both polarity strands (symbols are as reported in the legend to Fig. 2) and can be considered as a replicative module derived from a protoviroid that later captured the RNA sequences now forming the right and left terminal domains. According to this model, different sequences were captured in the left part of the molecule of the short and long FHVd-LRs.

In both the cases just described, RNA molecules of different sizes coexist in the same host and are likely derived from recombination events (Di Serio et al. 2006; Olmedo-Velarde et al. 2020), thus supporting a role of a jumping RNA polymerase in the evolution of these RNA replicons (Fig. 7).

### 5.3 A plant retroviroid-like element: from RNA to DNA

Several carnation accessions have been shown to contain a unique species of small RNA with close similarities to plant viroid and viroid-like satellites RNAs. In addition to its small size (275 nt), circular structure and ability to adopt HHRs in both polarity strands (Hernández et al. 1992), this carnation viroid-like RNA also exhibits one remarkable difference: it coexists with tandem repeats of a homologous DNA fused to sequences of either a pararetrovirus or the host (Daròs and Flores 1995; Hegedűs et al. 2001). This retroviroid-like element, so-called because the homologous DNA is presumably generated by the reverse transcriptase (RT), is vertically (but not horizontally) transmissible and not associated with any visible symptom. Moreover, the (+) HHR of the carnation viroid-like RNA is structurally more related to the HHR of a tandemly repeated 330-nt sequence scattered throughout the newt chromosomes (Epstein and Gall 1987) than to the HHRs reported in plants (Hernández et al. 1992; Daròs and Flores 1995). The existence of HHRs in carnation viroid-like RNA of both polarity strands is consistent with its RNA-RNA replication through a symmetric double rolling circle mechanism, while the lack of horizontal transmission suggests dependence on an initial input of transcripts from the DNA form.

More recently, several studies have revealed the presence of sequences ‘encoding’ HHR in a large number of genomes in the tree of life (de la Peña and García‐Robles 2010; Seehafer et al. 2011; Hammann et al. 2012; de la Peña, Ceprián, and Cervera 2020), thus expanding the initial discovery in the newt genome. Focusing on plants, comparative genomic analysis of more than 40 species revealed that the tandem HHR motifs are embedded within the sequence of a novel family of nonautonomous retroelements, the retrozymes, whose transcripts accumulate *in vivo* as abundant noncoding circular RNAs of the size (600–1,000 nt) expected for HHR-mediated self-cleavage and circularization (Cervera, Urbina, and de la Peña 2016). These circular RNAs adopt compact secondary structures *in silico,* and both (+) and (−) strands are detected *in planta,* suggesting that, like viroids and viroid-like satellite RNAs, they might undergo RNA-RNA replication through a rolling circle mechanism (Cervera, Urbina, and de la Peña 2016).

Although it has been proposed that HHR-containing viroids may have evolved from retrozyme circRNAs existing in plant transcriptomes, the obvious counter-argument is that retrozymes themselves could have originated from viroid RNAs (de la Peña and Cervera 2017). Given that carnation retroviroid-like DNA element has been found fused to sequences of a pararetovirus (encoding a RT) (Daròs and Flores 1995), we consider it more likely that retrozyme evolution has followed the same route as that of the retroviroid-like element, that is, by reiterated reverse transcription of the circular RNA form into DNA tandem repeats. Consistent with this view, typical retrotransposons, most of them ancient RNA viruses, have become inserted in their host genomes via reverse transcription.

### 5.4 Hepatitis delta virus and other deltaviruses are chimeric RNAs formed by ribozymatic and coding RNA sequences

The discovery of a novel antigen (the delta antigen, ∂Ag) in humans infected by hepatitis B virus (HBV) (Rizzetto et al. 1977) was the first step leading to the identification of hepatitis delta virus (HDV). The ∂Ag comigrated with a distinct fraction of HBV virions and with a small RNA (∼1700 nt) during gradient centrifugation. This finding suggested that such small RNA could be the genome of the ∂ agent (Rizzetto et al. 1980), thus representing the smallest virus genomic RNA, just five-fold larger than plant viroid RNAs (see, for a review, Flores, Ruiz-Ruiz, and Serra 2012). A subsequent report showing that (+) and (−) HDV RNAs were single-stranded circular molecules adopting rod-like secondary structures extended their similarity with viroids (Chen et al. 1986). Like a viroid, HDV replicates autonomously, with HBV providing only the envelope protein needed for transmission (Chen et al. 1986). In contrast to viroids, however, the unencapsidated antigenomic HDV RNA encodes two isoforms of the ∂Ag (Wang et al. 1986) via an ∼900 nt subgenomic RNA that contains a 5ʹ-cap and a 3ʹ-polyA tail (Gudima et al. 2000). HDV RNA replicates in the nucleus through a symmetric double rolling circle mechanism (Chen et al. 1986), in which the synthesis of the oligomeric RNA (+) and (−) intermediates is catalyzed by Pol II redirected to transcribe RNA templates just like in the family *Pospiviroidae* (Taylor et al. 1987). The replicative intermediates self-cleave cotranscriptionally via *cis*-acting HDV ribozymes, which are different from the HHRs (Kuo, Chao, and Taylor 1989; Ferré-D’Amaré, Zhou, and Doudna 1998; Chadalavada, Cerrone-Szakal, and Bevilacqua 2007). The resulting monomeric linear RNAs are circularized by a host enzyme (Reid and Lazinski 2000) other than the DNA ligase 1 used by PSTVd; that enzyme requires 5ʹ-phosphomonoester and 3ʹ-hydroxyl termini (Fig. 1), while those generated by the HDV ribozymes are 5ʹ-hydroxyl and 2ʹ,3ʹ-cyclic phosphodiester (Ferré-D’Amaré, Zhou, and Doudna 1998).

The modular structure of its antigenomic RNA provides a hint about the evolutionary origin of HDV. Like that of PLMVd and other pelamoviroids, HDV genomic RNA is composed of a viroid-like domain (∼300 nt) containing the ribozymes located in a terminal portion of the rod-shaped conformation. In contrast to viroids, however, this terminal domain is fused to a protein-coding domain. In one plausible scenario, the antigenomic HDV RNA may have evolved from a primordial self-replicating viroid (emerged in the RNA world) by the capture (in the protein/DNA world) of an mRNA encoding a ∂Ag-like protein, thereby enhancing the survival of the fusion product (Brazas and Ganem 1996; Robertson 1996). The widespread presence of HDV ribozymes in eukaryotic genomes (Webb et al. 2009) has also led to an alternative scenario in which HDV RNA has evolved from cellular RNAs that, after having been processed into circular forms, would have gained the ability to replicate autonomously (Salehi-Ashtiani and Szostak 2001; Taylor and Pelchat 2010; Taylor 2014).

New metatranscriptomic data provided yet another unexpected twist: HDV-like agents (deltaviruses) have been reported in multiple organisms, including birds (Wille et al. 2018), snakes (Hetzel et al. 2019; Szirovicza et al. 2020), fish, amphibians and invertebrates (Chang et al. 2019), and rodents (Paraskevopoulou et al. 2020). Most importantly, enveloped viruses distinct from HBV induce *in vivo* dissemination of HDV (Perez-Vargas et al. 2019) and HDV-like agents (Chang et al. 2019). These results suggest that HDV-like agents may have been associated with animal hosts throughout the evolutionary history of metazoans (Chang et al. 2019), thereby arguing against any hypothesis for HDV origin based upon the singularity of the HDV/HVB. As expected, the ribozyme motifs in most of these HDV-like circRNAs (Wille et al. 2018; Hetzel et al. 2019; Paraskevopoulou et al. 2020; Szirovicza et al. 2020) are found in locations similar to those of the human deltavirus.

Of particular interest in this regard are HDV-like circRNAs identified in amphibians and termites (Chang et al. 2019), which contain not HDV-like ribozyme motifs but rather HHRs in either polarity strand (de la Peña, Ceprián, and Cervera 2020; de la Peña et al. 2021). This remarkable finding suggests that the HDV-like circRNAs present in amphibians and termites may have resulted from the fusion of a primordial self-replicating viroid (different from that of HDV) and an mRNA encoding a ∂Ag-like protein, thus widening and reinforcing the modular origin of deltavirus antigenomic RNAs. HDV and other HDV-like agents display an unusual type of parasitism; while replicating autonomously, they depend on helper viruses for transmission. Remarkably, at least in one instance, the HDV-like agent seems not to require helper virus coinfection (Paraskevopoulou et al. 2020), thus behaving like a viroid.

## 6. General considerations for the origin of viroids: the RNA world versus other scenarios

The hypothesis that viroids are host-derived, in the past considered unlikely mainly due to the lack of any traceable sequence identity between the genomes of viroids and their hosts, has been recently revived (de la Peña and Cervera 2017; Catalán et al. 2019; Jain et al. 2020). Based on numerical simulations and taking into consideration specific constraints, Catalán et al. (2019) showed that the selection of RNAs with the typical rod-like shapes characteristic of most viroids would be unlikely if evolution started with relatively long sequences, for example, cellular, viral or sub-viral RNA. Instead, gradual addition of nts to minimal circular RNAs and independent acquisition of functional modules trigger the emergence of larger RNAs with a structure analogous to viroids (Catalán et al. 2019). Although the authors identify some small cellular RNAs as potential seeds of the process, they also admit that ‘in a prebiotic scenario, random RNA sequences could have fulfilled these minimal conditions, such that viroid-like replicons could have easily emerged in a precellular context’.

**Figure 8.**
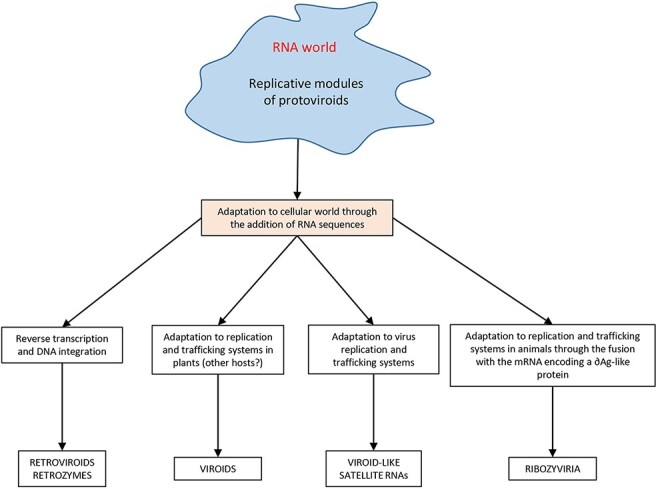
Hypothesis on the origin of viroids, viroid-like RNAs, retroviroids and retrozymes, and the members of the realm ribozyviria (hepatitis delta and delta-like viruses) through a modular evolutionary scenario based on the fusion of replicative modules of protoviroids, deriving from the RNA world, and RNAs of cellular origin.

Importantly, the evolutionary model proposed by Catalán et al. (2019) also applies to protoviroids: such entities could have seeded the process during their early adaptation to the cellular environment by providing the necessary replicative module and/or functional motifs (i.e. ribozymes), likely originated in the RNA world (Fig. 8). On the other hand, the bacteriophage T7 RNA polymerase has been recently shown to generate and amplify diverse RNA sequences *in vitro* through partial instruction from DNA seeds, thus suggesting that a similar process could also be regarded as a possible original step of the viroid genesis (Jain et al. 2020). Whether the reported enzymatic activities may also exist *in vivo*, in a more competitive and complex cellular environment where the polymerases likely preferentially target their physiologic substrates, is not known.

Another question pertinent in this context is whether HHRs evolved once in the RNA world or several times in other scenarios. *In vitro* evolution, involving the transcription of DNA random pools, identification of self-cleaving RNAs, reverse transcription of the corresponding full-length transcripts, and PCR amplification, followed by subsequent rounds of selection/amplification, has been taken as evidence supporting multiple origins for HHR (Salehi-Ashtiani and Szostak 2001). The very simple conditions of the *in vitro* self-cleavage assay, an aqueous solution buffered around neutrality containing low levels of divalent ions, are more reminiscent of those in the RNA world (where random RNA pools may have conceivably existed) than to those present in complex cellular habitats, which are divided into membranous organelles crowded with multiple proteins that outperform RNA in their chemical versatility. Moreover, the replicative catalytic module of protoviroids seems a particularly good candidate to have emerged in the RNA world, considering that the three catalytic activities mediating rolling circle replication (i.e. RNA polymerization, cleavage, and ligation) can be provided by ribozymes, thus making this replication mechanism plausible in a protein-free world (Flores et al. 2014). Interestingly, a ligation-based modular evolution has been proposed to consider the increasing functional complexity in the RNA world (Briones, Stich, and Manrubia 2009).

Even those critics of the emergence of viroids in the RNA world admit that ‘among all known replicons, viroids come the closest to what one would envisage as a vestige of the RNA world’ (see reviewers’ comments in Diener 2016). One critic focused on the fact that viroids are specific to plant cells, with a host range restricted to angiosperm. For the family *Avsunviroidae,* whose members replicate in chloroplasts through a mechanism mediated by HHRs (the fingerprint of the RNA world), one might reasonably expect to find some vestiges of such RNAs in cyanobacteria, from which chloroplasts evolved by endosymbiosis (Margulis 1993). Indeed, according to Chela-Flores, ‘if shown to be associated with cyanobacteria, viroids could have been present during the major part of the duration of life on Earth’. Definitive data regarding such association are so far lacking, although metagenomic studies on these prokaryots may have not been sufficiently deep and, to the best of our knowledge, have not searched for small circular RNAs. Perhaps, protoviroids were only present in cyanobacteria lineages with no living descendants.

While able to support the replication of some viroids, plant chloroplasts seem hostile to viruses since none has been reported in this organelle, with only some partitivirus-like dsRNA replicons having been associated with chloroplasts of a green alga (Koga, Horiuchi, and Fukuhara 2003). In sharp contrast, present-day cyanobacteria support three families of dsDNA viruses (i.e. *Myoviridae, Siphoviridae*, and *Podoviridae*) (Lavigne, Molineux, and Kropinski 2012; Chénard, Wirth, and Suttle 2016). In the course of endosymbiosis, a large fraction of the cyanobacteria genome was transferred to the nucleus of the holosymbiont, which, in turn, encodes and targets to the chloroplast (via transit peptides) the vast majority of proteins found in this organelle. However, this process has not been uniform as illustrated for instance by gene *accD* encoding a subunit of the acetyl-CoA carboxylase, which is located in the plastid genome of most angiosperms, whereas in a few families, it is a nuclear gene (Magee et al. 2010). Perhaps, some viroids were also lost during this major transition or during the subsequent rearrangements. Indeed, viroids are confined not only to plants but to particular plants species, with other plants being resistant to infection possibly because, during their evolution, some lineages lost critical components to sustain this process.

A characteristic feature of members of the family *Avsunviroidae* is their narrow host range, restricted to those where they were initially reported (i.e. ASBVd in avocado, PLMVd in peach, and CChMVd in chrysanthemum), with high-throughput sequencing of other plant species having failed so far to retrieve sequences of these but not of other viroids, including a novel apscaviroid unexpectedly infecting a monocot (an orchid) (Yang et al. 2020). Such observations suggest an old viroid/plant coevolution for members of the family *Avsunviroidae*. Although their very high mutation frequency (Gago et al. 2009) would suggest an ability to infect additional hosts, this is not the case, even in very favorable experimental settings. The ultimate reason remains unknown, as also does how ASBVd got to colonize specifically avocado, and the other members of the family *Avsunviroidae* their corresponding natural hosts.

Do members of *Avsunviroidae* and *Pospiviroidae* families share a common origin? Phylogenetic analyses do not answer this question, but certain hints are consistent with a link: the right terminal domain of HSVd contains a hammerhead-like structure, and the lower strand of the CCCVd CCR may contain a self-cleavage site. Moreover, PSTVd and ASBVd use during replication Pol II and NEP, respectively, with HDV RNA behaving like PSTVd. It is remarkable that viroids and this viroid-like RNA have found the same solution: to divert DNA-dependent RNA polymerases to transcribe RNA templates. The crystal structure of Pol II bound to a scaffold (Lehmann, Brueckner, and Cramer 2007) is consistent with the possibility that a Pol II ancestor could replicate primitive RNA genomes like the precursors of viroid and viroid-like RNAs during their transition from the RNA world to the protein/DNA world. A similar framework is possible for DNA ligase 1, which acts as an RNA ligase in the circularization of the monomeric linear PSTVd (+) RNA with 5ʹ-phosphomonoester and 3ʹ-hydroxyl termini. Intriguingly, the T4 phage RNA ligase 2 requires the same termini and functions through a mechanism similar to DNA ligase 1 (Ho and Shuman 2002; Wang, Schwer, and Shuman 2006), and an ancestral catalytic module mediating RNA repair has been proposed as a common precursor for these enzymes (Shuman and Lima 2004). The ability of DNA ligase 1 to act on viroid RNAs may just reflect the original template of its precursor enzyme.

In contrast, in the family *Avsunviroidae,* where the 5ʹ-hydroxyl and 2ʹ,3ʹ-cyclic phosphodiester termini generated by HHRs would have been ligated initially by the HHR, this ribozyme activity would have been later replaced in the protein/DNA world by a chloroplastic tRNA ligase specific for the same termini (Gas et al. 2007; Nohales et al. 2012b). These termini would be brought in close proximity and orientation by the ancestor protoviroid secondary structure. Therefore, the compact circular secondary structure itself appears to be a trait of the RNA world, partly resulting from selfcopy by a jumping RNA polymerase and partly from the capture of domains/HPs. Such an RNA-based strategy would have allowed viroids to survive without the need for a second module involved in virion assembly as proposed for RNA viruses (Krupovic and Koonin 2017; Wolf, Kazlauskas, and Iranzo et al. 2018; Krupovic, Dolja, and Koonin 2019). Moreover, such an ancient structural feature of protoviroids that is maintained in viroids—which accumulate *in planta* as free RNAs unprotected by tightly bound host proteins (López-Carrasco and Flores 2017a,b)—besides providing resistance to chemical (nucleases) and physical (UV irradiation) damage is also more robust to mutation (Elena, Gómez, and Daròs 2009; Catalán et al. 2019).

The relatively recent appearance of viroid diseases has been likely facilitated by modern agriculture, including: (1) vast expanses of genetically identical plants (monocultures) close to wild ecosystems that may be reservoirs of viroids, usually latent in the latter but potentially pathogenic for the former, (2) international trade of propagation material, and (3) use of diverse sources of germplasm in plant breeding, which if infected with a vertically transmissible viroid may spread it into new areas (Diener 1995). Additionally, in the case of woody plants, such as citrus and grapevine, their vegetative propagation together with a long productive life during which they may be repeatedly exposed to viroid infections (through pollen, grafting, and pruning tools) would be expected to facilitate viroid spread and recombination, resulting in the emergence of new viroids.

## 7. Conclusions

Based on the totality of current evidence, we believe that there are still good reasons to keep alive the hypothesis that viroids may be relics of precellular evolution (Diener 1989; Flores et al. 2014). An evolutionary scenario based on the fusion of the replicative modules of protoviroids, likely generated in the RNA world, with RNAs of cellular origin could explain the origin not only of viroids but also of the other viroid-like catalytic RNAs present in plants and animals, thus providing a parsimonious and unified model for their emergence and evolution in the cellular environment (Fig. 8). Unbiased metagenomic analyses have unveiled the astonishing diversity of the biosphere, in particular, that of the prokaryotic and virus worlds where only the tip of the iceberg may now be visible. We expect that additional studies will play a major role in shedding light on viroid origin. The discovery of several deltaviruses, some of which have HHRs instead of HDV-like ribozymes, is one example of the lesson to be learnt. Extension of such approaches to wild counterparts of cultivated plants and to nonplant ecosystems should help to clarify whether viroids and related RNAs are truly survivors of the RNA world, a key question in the search of the first replicons to emerge on our planet. Meanwhile, other scenarios should also be entertained.
